# Bone and Soft Tissue Sarcoma

**DOI:** 10.3390/cancers12092609

**Published:** 2020-09-12

**Authors:** Antonio Ruggiero

**Affiliations:** Pediatric Oncology Unit, Fondazione Policlinico Universitario A.Gemelli IRCCS, Universita’ Cattolica del Sacro Cuore, Largo A. Gemelli 8, 00168 Rome, Italy; antonio.ruggiero@unicatt.it; Tel.: +39-06-3015-5165; Fax: +39-06-305-2751

Bone and soft-tissue sarcomas are relatively rare tumors both in children and adults. The progress which has been achieved in the diagnosis and treatment of bone and soft tissue sarcomas is a history of remarkable success. From being an almost lethal disease 40 years ago, more than 60% of patients can now be offered treatment with cure as the most likely outcome [1]. This progress has been obtained as the result of basic research and the testing of new knowledge through cooperative multi-institutional and multinational clinical trials.

These tumors may be a diagnostic challenge for the clinicians taking into account the different tumor entities, the rareness of these tumors, and the considerable morphological heterogeneity which can characterize a single tumor entity.

Although histologic features alone appear to be conclusive for some bone and soft tissue tumors, immune-histochemistry continues to play a fundamental diagnostic role for most mesenchymal tumor types. In addition, the discovery of recurrent genomic alterations in many mesenchymal tumors has added important biologic insights and expanded the spectrum of some diagnostic subgroups (i.e., CIC-rearranged and BCOR-rearranged sarcoma) [2].

Based on the evidence that more than half of the soft-tissue tumors and about 25% of the bone tumors harbor recurrent genetic alterations, the identification of the molecular anomalies should be part of the diagnostic workup as they could represent not only a diagnostic tool but could permit the identification of potential targets for drug therapy [3,4]. 

Surgical excision has historically been the first therapeutic approach for the therapy of bone and soft-tissue sarcomas although it is noncurative in metastatic and large infiltrating tumors. Radiotherapy has been added in order to augment the local control. Although cytotoxic chemotherapy has made a fundamental contribution to the treatment of many sarcomas, classic treatment regimens can carry some significant side effects while outcomes have plateaued and as long as new drugs are utilized, it is possible that new adverse effects will be registered in the future [5,6,7,8].

This issue contains three original research reports examining novel targets in preclinical models (*n* = 2) and patients (*n* = 1).

Mattei et al. focused their attention on dedifferentiated liposarcomas (LPS) [9]. These tumors carry a poor prognosis for the high risk of recurrence due to the risk of metastases dissemination and of incomplete resection when arising into the retroperitoneal region. While dedifferentiated LPS are characterized by poor prognosis due to their aggressive behavior in comparison to well-differentiated LPS, both tumors present MDM2 and CDK4 amplification but dedifferentiated LPS are also characterized by an accumulation of nonspecific genomic aberrations, potential prognostic and therapeutic tools for new therapies. In this context, Aurora kinases A (AURKA) and B genes were significantly linked to poor prognosis. In their study, Mattei et al. observed a different correlation between overall survival, progression-free survival, metastatic recurrence and AURKA and AURKB mRNA expression. Then, they confirmed that AURKA and AURKB are heretogeneously expressed in nine sarcoma cell lines while both have low expression in normal muscle tissue when compared to sarcoma cell lines. They concluded their analysis with evidence that a correlation is present between AURKA and AURKB mRNA overexpression and a low metastasis-free survival and the observation that AuroraKinase inhibition operated by AMG900 could be a promising treatment for LPS.

Goetz et al. retrospectively evaluated the prognostic impact of surgical margins in 192 patients with primary undifferentiated pleomorphic sarcomas of the extremities [10]. Almost all patients (93.2%) obtained negative surgical margins but distant metastases were detected in 27.1% of patients during the follow-up time. The 2-year local recurrence-free survival (LRFS) and overall survival (OS) registered a significant difference between R0-resected primary tumors and R1/R2-status highlighting the prognostic impact of negative margins. Adjuvant radiotherapy significantly improved LRFS (5-year: 67.6% vs. 48.4%; *p* < 0.001) and OS (5-year: 82.8 vs. 61.8; *p* = 0.016) by reducing the risk of local failure. In their multivariate analysis, negative surgical margins and adjuvant radiotherapy appeared to influence the OS and to be independent prognostic factors. Therefore, microscopic negative margins appear to have a remarkable impact on LRFS and OS; patients who obtained microscopic negative surgical margins had a significantly better LRFS and OS than patients with positive margins, suggesting that a more aggressive surgical approach should be attempted in order to obtain wider negative margins.

In their exploratory study, Reader et al. characterized the expression of class III β-tubulin and EP4 in gynecologic leiomyosarcoma (LMS) [11]. LMS is an aggressive gynecologic sarcoma with a poor prognosis in advanced stages. Its 5-year disease-specific survival can range from 76% (stage I) to <29% (stage IV). Regarding its treatment, gemcitabine plus docetaxel is in fact one of the most chemotherapeutic regimens adopted with an overall response rate of 27%.

In a total of 29 cases of uterine smooth muscle tumors, they analyzed the protein expression of class III β-tubulin and EP4 in order to test that LMS express higher levels of class III β-tubulin and EP4 than those with indeterminate or benign nature and that their expression can be correlated with resistance to taxanes treatment. The overexpression of class III β-tubulin was detected in many malignant tumors and was correlated with taxane resistance.

They concluded that class III β-tubulin and EP4 expression in LMS can be a valid candidate for identifying patients that may be at risk of resistance to standard chemotherapeutic regimens. In addition, class III β-tubulin and EP4 expression may be helpful to detect patients as candidates for different cytotoxic regimens through EP4 inhibition.

In conclusion, despite the recent data on the treatment and on the molecular features of bone and soft-tissue tumors, sarcomas remain a challenging area with many concerns about the prognosis and unmet therapeutic needs. This Special Issue aims to contribute to the further understanding of the biology of these aggressive tumors and provides specific biomarkers for the development of new therapies in order to improve the survival and quality of life of patients affected by these malignant tumors.
